# Prevalence of HIV infection among siblings of HIV positive children in Calabar, Nigeria

**DOI:** 10.11604/pamj.2019.32.179.16837

**Published:** 2019-04-11

**Authors:** Sunday Oteikwu Ochigbo, Chimaeze Torty, Maxwell Anah

**Affiliations:** 1 Faculty of Medicine, Department of Paediatrics, University of Calabar, Calabar, Nigeria; 2 Department of Paediatrics, University of Calabar Teaching Hospital, Calabar, Nigeria

**Keywords:** Siblings, HIV, health, family

## Abstract

**Introduction:**

Early diagnosis and treatment of paediatric HIV is key as mortality of untreated patients is very high in the first two years of life, and reaches 80% by four years. Case finding efforts for children especially outside Prevention of mother-to-child transmission (PMTCT) is inadequate. Targeting siblings of index HIV-exposed and infected children is an important way of improving identification and enrolment into care thereby reducing paediatric mortality. The study therefore aimed to determine the prevalence of HIV infection among siblings of HIV positive children in care in Calabar.

**Methods:**

This descriptive cross-sectional study was conducted among children aged six weeks to 15 years who are siblings of HIV positive children receiving care. Parental consent and child assent were obtained, the children were tested for HIV at their homes irrespective of their prior test results. Ethical clearance certificates were obtained from the health institutions.

**Results:**

Siblings of 401 index patients were tested for HIV, four were positive giving a prevalence rate of 1%. Three hundred and sixty-seven 367(91.5%) had been tested previously while 34(8.5%) never had HIV test. Among the siblings who were HIV positive, 1(0.3%) was a male while 3(0.7%) were females. There were more HIV positive siblings in the 11-15 years age group.

**Conclusion:**

All the four HIV positive siblings were from the lower socioeconomic class (p=0.022). The routine screening of siblings of HIV positive children should be sustained with focus on adolescents from the lower socioeconomic class. This will improve early identification and enrolment into care thereby reducing paediatric mortality.

## Introduction

According to the Joint United Nations Programme on HIV/AIDS (UNAIDS) Gap report of 2012, 19 million out of the 35 million living with HIV are unaware of their sero-status. In Sub-Saharan Africa, only 48% of adults living with HIV know their status [1, 2]. In Nigeria, only 35% of people living with HIV know their status [3]. These figures may be higher in children. Identifying and diagnosing children, as HIV infected is the first step in the continuum of paediatric care and treatment, yet despite the availability of life-saving treatment, many HIV infected children are never offered any HIV diagnostic test [4]. Even in the phase of Provider Initiated Testing and Counseling (PITC), diagnosis and treatment gaps still remain a major barrier to HIV care.

Early diagnosis and treatment of paediatric HIV is key as mortality of untreated patients is very high in the first two years of life and reaches 80% by four years [5]. Paediatric antiretroviral therapy (ART) coverage is only 20.7% leaving a gap of 80% [6]. Central to provision of treatment and support to families affected by HIV is knowledge of the HIV status of all family members and linkage to appropriate prevention, treatment and care services [1]. Case finding efforts for children especially outside PMTCT is also inadequate [7]. There is a need to scale up uptake of paediatric HIV testing services beyond the provider-initiated efforts. The present strategy of waiting to test only those children presenting to health facilities often with advanced clinical disease is inadequate [8]. Testing other family members has been identified to specifically increase family diagnosis of HIV infection [9]. Targeting siblings of index HIV-exposed/infected children is an important way of improving identification and enrolment into care thereby reducing paediatric mortality [9]. The study therefore aimed to determine what proportion of siblings of HIV infected children actually infected with HIV.

## Methods

This descriptive cross-sectional study was conducted from 6th November 2017 to 23rd March 2018 in two principal health facilities offering PMTCT/Paediatric HIV services in Calabar, Nigeria. These facilities included the University of Calabar Teaching Hospital (UCTH) and General Hospital, Calabar. The UCTH is a 600-bed tertiary health facility while General Hospital, Calabar is a secondary healthcare center with 100-bed capacity. The average number of HIV positive children in care in these two health facilities are 100 and 145 in UCTH and General Hospital Calabar respectively. All siblings aged 6 weeks to 15years of Index patients attending the HIV clinic that met the inclusion criteria were traced and screened for HIV infection.

A “sibling” here is defined as a child from the same biological parents as the index patient. Addresses and phone numbers were used to contact the parents /guardians and those with siblings were identified. The purpose of the study was explained to each parent/guardian and a convenient date and time for home visit was scheduled. Parental consent and child assent were obtained, the siblings aged 6weeks to 15years in each family were tested in their homes irrespective of the prior test result. In families with more than one child receiving HIV care, the first child on the clinic register was used as the index to reach out to other siblings. Those found to be positive and not on treatment were linked to a health facility for enrolment into treatment and care. Those who objected to home visit but consented to HIV test for their children were screened at the facility where the index patient received Anti-retroviral drugs (ARVs). Children whose parents or guardian refused consent were not denied services. Siblings of HIV positive children from both health facilities younger than six weeks or older than 15 years and children whose parents or guardians declined consent were excluded from the study. Ethical Clearance Certificates were obtained from the Health Research Ethics Committee of the University of Calabar Teaching Hospital and the Ministry of health, Cross-Rivers state.

Data analysis was done using SPSS (Statistical Package for Social sciences) version 21 statistical software. Descriptive statistics like frequency, histograms and percentages were generated for siblings of HIV positive children previously non-tested and previously tested for HIV. In addition, Chi-square test was used to examine the relationship between the socio-demographic characteristics and HIV results of the siblings. Fisher exact test was used to examine the relationship between variables when the expected frequency is less than 5. P-value less than 0.05 of the test measured was considered statistically significant.

## Results

**Figure 1:**
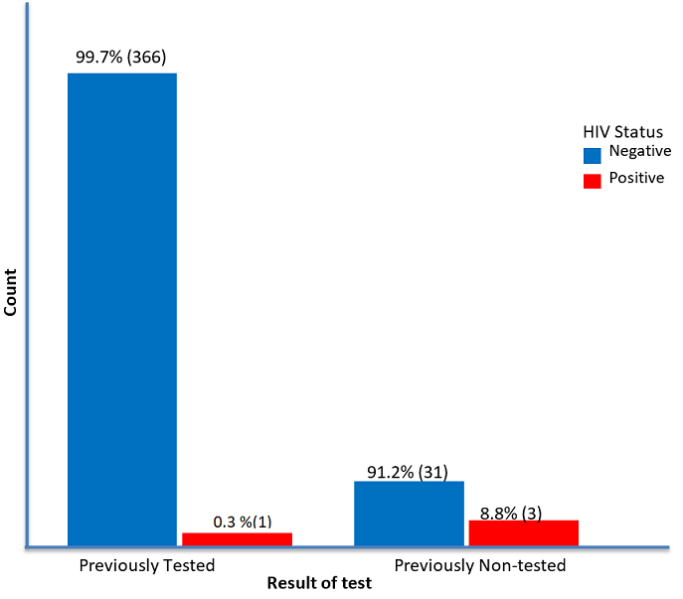
Prevalence of HIV infection in previously non-tested and previously tested siblings of HIV positive children in Calabar

Four hundred and one (401) children were encountered in 212 families. Of the 401 siblings studied, four-tested HIV positive, giving an HIV prevalence rate of 1% (Figure 1).Three hundred and sixty-seven 367(91.5%) had been previously tested for HIV while 34(8.5%) never had HIV test. Among those that had a previous HIV test, one child (0.3%) tested positive while 366(99.7%) tested negative. This child aged 14years had been previously diagnosed with HIV infection before this study but was not enrolled into care. The 366 that tested negative knew their status prior to this study and there was no seroconversion. However, out of the 34 that had HIV test for the first time, 3(8.8%) and 31(91.2%) tested positive and negative respectively (Figure 1). Among siblings who were HIV positive, 1(0.3%) was a male while 3(0.7%) were females. The difference was not statistically significant (p=0.625). Regarding age group, there were more HIV positive siblings in the 11-15 years age group. The difference was not significant statistically (p=0.696). All those who were HIV positive belonged to the 1-4 birth order (1.0%) and were mostly in household with 1-4 children. There was no statistically significant difference between HIV result, birth order (p=1.000) and number of children in household (p=0.514) (Table 1). All the four HIV positive siblings were from the lower socioeconomic class, this was statistically significant (p=0.022). Interestingly, all those that tested positive in the study were older than their index siblings.

**Table 1:** Relationship between socio-demographic characteristics and HIV results of study participants (N = 401)

Variables	HIV results			Chi-square test	P-value
	Positive (%)	Negative (%)	Total (%)		
**Sex**					
Males	1(0.3)	191(47.6)	192(47.9)	Fisher’s exact	0.625
Females	3(0.7)	206(51.4)	209(52.1)		
**Age groups**					
≤5 years	1(0.2)	151(37.7)	152(37.9)	Fisher’s exact	0.696
6-10 years	1(0.2)	135(33.7)	136(33.9)		
11-15 years	2(0.5)	111(27.7)	113(28.2)		
**Birth order**					
1-4	4(1)	384(95.8)	388(96.8)	Fisher’s exact	1.000
>4	0(0.0)	13(3.2)	13(3.2)		
**Children in household**					
1-4	3(0.7)	332(82.8)	335(83.5)	Fisher’s exact	0.514
>4	1(0.3)	65(16.2)	66(16.5)		
**Socioeconomic class**					
Upper Class	0(0.0)	97(24.2)	97(24.2)	Fisher’s exact	0.022*
Middle Class	0(0.0)	176(43.9)	176(43.9)		
Lower class	4(1.0)	124(30.9)	128(31.9)		

* Statistically significant

## Discussion

The prevalence of HIV infection among siblings of HIV positive children in Calabar was observed to be 1%. There is paucity of data on prevalence of HIV among siblings of HIV positive children. However, the result of the current study was similar to the prevalence of 0.9% described by Ng'eno *et al.* [10] in a community based cross-sectional study in Kenya. The result of this study was lower than the HIV prevalence of 4.2% by Venn *et al.* [11] among infants attending immunization clinics. The finding of this study was also lower than the prevalence of 5.3% reported by Ntia *et al.* [12] in a prospective study of under-5 children admitted with diarrhea. These hospital-based studies [11, 12] were both done in the same locality (Calabar, South-south Nigeria) as the current study, suggesting a difference in the trend of HIV positivity rates in this locality between the general population and siblings of HIV positive children. The former study [11] dealt with asymptomatic children at immunization centers while the later studied symptomatic children with diarrhea and it was not surprising with the observed prevalence. The low prevalence in this study could be attributed to increased facility coverage of PMTCT in Calabar in the last few years as a lot of work has been done especially by government and non-governmental organizations in improving access to HIV diagnosis and care.

This prevalence was much lower than the 10% reported by Ogunbosi *et al.* [13] in Ibadan South-west Nigeria, and the 26.9% in Brazil reported by Alberto *et al.* [14] The Ibadan study was hospital based as compared to this study which was community based. Furthermore, their study [13] site is a referral center and this may have contributed to the high prevalence noted. Most hospital-based studies [15-17] had higher prevalence rates as they mostly target children with suggestive clinical features. The study in Brazil [14] was similar to our study, tracing siblings of HIV positive children under care. This was thought to be due to repeated MTCT. The lower prevalence in the current study could be attributed to the fact that it was done when most of the mothers of the index HIV positive children were already on ART with most of the tested siblings having benefitted from PMTCT unlike in the Brazilian study which was conducted at a time when more than half of the HIV positive mothers were HAART naïve and therefore the higher risk of transmission to their children. Similarly, Yumo *et al.* [18] observed a prevalence of 18.2% in an active search for paediatric HIV by systematic screening of the children of HIV infected parents in Cameroun. Unlike the study by Yumo *et al.* [18] that used parents as index cases to search for and test their children, this study used index paediatric cases to trace their siblings therefore the patients in care were not part of the calculated prevalence thus the lower value of the siblings prevalence than the Cameroonian study. Despite the policy of testing for HIV in family members of any index patient with HIV in both the WHO and Nigerian National guidelines, implementation of this practice has been poor.

HIV is a disease that disproportionately affects those with socioeconomic status [6]. As such social inequalities may result in disparities in HIV health outcomes. Researches have suggested that a person's socioeconomic standing may affect his or her likelihood of contracting HIV and developing AIDS [19-21]. Our study also corroborated the above findings indicating that 100% of the siblings who turned to be HIV positive were from the lower socioeconomic status. Adejuyigbe *et al.* in Ife, Nigeria [22] also reported high correlation between HIV and AIDS, poverty and low maternal education.

## Conclusion

The low prevalence rate of 1% observed therefore suggest that screening siblings of HIV positive children might not be very reliable channel for identification of paediatric HIV infections. In addition, focus should be on siblings from the low socioeconomic class.

**Limitations**: In view of the small sample size, it might be difficult to generalize the findings, however further studies with larger sample size are recommended.

### What is known about this topic

- Evidences have shown that there is high HIV prevalence through active search for paediatric HIV by systematic screening of HIV infected parents;
- Early identification of HIV and enrollment reduces paediatric morbidity and mortality.

### What this study adds

- The prevalence of HIV infection among siblings of HIV positive children in Calabar was 1%;
- Siblings from the lower socioeconomic class could have higher infection rate.

## Competing interests

The authors declare no competing interests.

## References

[1] UNAIDS (2014). The Gap report: Beginning of the end of the HIV/AIDS epidemic.

[2] UNAIDS Close the gap: close the education gap, World AIDS day 2014. 2014.

[3] UNAIDS (2017). Joint United Nations Programme on HIV/AIDS (UNAIDS).

[4] Kellerman S, Essajee S (2010). HIV testing for children in resource-limited settings: what are we waiting for. PLoS Med.

[5] Newell M, Coovadia H, Rollins N, Gaillard P, Dabis F (2004). Mortality of infected and uninfected infants born to HIV-infected mothers in Africa: a pooled analysis. Lancet.

[6] WHO,UNAIDS (2015). Access to Antiretroviral Therapy in Africa: status Report on Progress towards the 2015 Targets.

[7] Kellerman S, Essajee S (2010). HIV testing for children in resource-limited settings: what are we waiting for. PLoS Med.

[8] Ahmed S, Kim MH, Sugandhi N, Ryan P, Rachel S, Mamadou O (2013). Beyond early infant diagnosis: case finding strategies for identification of HIV-infected infants and children. AIDS.

[9] Busari O, Adeyemi A, Jimoh A, Nakayima M, Fasae A (2012). Routine HIV testing of family members of hospitalized patients in Nigeria. Prev Med Bull.

[10] Ng'eno B, Mwangi A, Ng'ang'a L, Andrea K, Anthony W, Irene M (2014). Burden of HIV infection among children aged 18 months to 14 years in Kenya: results from a nationally representative population-based cross-sectional survey. J Acquir Immune Defic Syndr.

[11] Venn J, Ochigbo SO, Anah M, Asindi A (2016). HIV sero-prevalence among infants attending immunization centres in Calabar metropolis Cross River state, Southern Nigeria. Int J Child Health Nutr.

[12] Ntia H, Anah M, Eyong K, Ikpeme O (2012). HIV Infection in hospitalized under-5 children with acute watery diarrhoea in Calabar, Nigeria. Nig J Paediatr.

[13] Ogunbosi BO, Oladokun RE, Brown BJ, Osinusi KI (2011). Prevalence and clinical pattern of paediatric HIV infection at the University College Hospital, Ibadan, Nigeria: a prospective cross-sectional study. Ital J Paediatr.

[14] RAmos AN, Matida HL, Hearst N, Oliveira FA, Heukelbac J (2012). High occurrence of HIV-positive siblings due to repeated mother-to-child transmission in Brazil. AIDS Care.

[15] Alikor EN (2005). Trend of HIV sero-positivity in a tertiary health institution in the Niger Delta region of Nigeria. Afr J Health Sci.

[16] Bugaje MA, Aikhionbare HA (2006). Paediatric HIV/AIDS seen at Ahmadu Bello University Teaching Hospital Zaria, Nigeria. Ann Afr Med.

[17] Edochie Y (2016). Pathophysiology of HIV/AIDS- Wikipedia, the free encyclopedia.

[18] Yumo H, Angwafor S, Ayuk E, Ndang C (2008). Scaling up HIV in paediatric care and treatment in resource limited settings. Lessons learned from active search for paediatric AIDS approach in a rural district in Northwestern cameroun.

[19] Pellowski JA, Kalichman SC, Matthews KA, Adler N (2013). A pandemic of the poor: social disadvantage and the US HIV epidemic. Am Psychol.

[20] Buot M-LG, Docena JP, Ratemo BK, Bittner MJ, Burlew JT, Nuritidinov AR (2014). Beyond race and place: distal sociological determinants of HIV disparities. PLoSONE.

[21] Latkin CA, German D, Vlahov D, Galea S (2013). Neighborhoods and HIV: asocial ecological approach to prevention and care. Am Psychol.

[22] Adejuyigbe EA, Fasubaa OB, Onayade AA (2004). Sociodemographic characteristics of HIV-positive mother-child pairs in Ile-Ife, Nigeria. AIDS Care.

